# Prevalence and patterns of dietary supplement use and potential drug interactions among older adults in Saudi Arabia

**DOI:** 10.3389/fphar.2025.1654337

**Published:** 2025-10-08

**Authors:** Wael Y. Khawagi, Majd M. Alghamdi, Laila M. Alfalqi, Atheer A. Alzahrani, Sara K. Alotaibi, Joud J. Alsuwat, Fahad H. Baali, Wadia S. Alruqayb, Sofa D. Alfian, Abdullah A. Alshehri

**Affiliations:** ^1^ Department of Clinical Pharmacy, College of Pharmacy, Taif University, Taif, Saudi Arabia; ^2^ College of Pharmacy, Taif University, Taif, Saudi Arabia; ^3^ Department of Pharmacology and Clinical Pharmacy, Faculty of Pharmacy, Universitas Padjadjaran, Jatinangor, Indonesia; ^4^ Center of Excellence for Pharmaceutical Care Innovation, Universitas Padjadjaran, Jatinangor, Indonesia; ^5^ Center for Health Technology Assessment, Universitas Padjadjaran, Jatinangor, Indonesia

**Keywords:** dietary supplements, older adults, drug interactions, Saudi Arabia, patient safety

## Abstract

**Introduction:**

The widespread use of dietary supplements (DSs) among older adults poses a potential risk for adverse interactions with prescribed medications, particularly in populations with multiple chronic conditions. In Saudi Arabia, where DSs commonly used, with limited data on the prevalence and risks of such interactions. This study aimed to assess the prevalence and patterns of DS use and evaluate potential drug–supplement interactions and their predictors among older adults.

**Methods:**

A cross-sectional study was conducted between November 2024 and March 2025 among outpatients aged ≥60 years. Data were collected through face-to-face interviews using a structured questionnaire. Drug–supplement interactions were assessed using Stockley’s Drug Interactions database. Data were analyzed using descriptive statistics, chi-square tests, and logistic regression in Stata.

**Results:**

A total of 293 older adults participated in the study, of whom 245 (83.6%) reported using DSs, with vitamin D being the most commonly used (40.0%). Female gender (OR = 2.17; 95% CI: 1.16–4.07) and hypertension (OR = 2.35; 95% CI: 1.24–4.47) were significantly associated with DS use. Among the 232 participants who used both DS and prescribed medications, 40.1% had at least one potential drug–supplement interaction, while 10.3% experienced at least one supplement–supplement interaction. The most frequently implicated supplements were nicotinic acid, vitamin D, and omega-3 fatty acids. Diabetes (OR = 3.67; 95% CI: 2.06–6.54) and hypertension (OR = 2.34; 95% CI: 1.34–4.09) were significant predictors of potential interactions.

**Conclusion:**

DS use is highly prevalent among older adults in Saudi Arabia, with a substantial proportion exposed to potential drug–supplement interactions. These findings highlight the importance of incorporating DS use assessment into routine clinical care for older adults and underscore the need for improved patient education and medication reconciliation practices to enhance medication safety.

## 1 Introduction

The use of dietary supplements (DSs) is increasing rapidly worldwide, as individuals increasingly turn to these products to prevent or manage various health conditions across different healthcare systems (U.S. Food and Drug Administration, 2005; World Health Organization, 2004). DSs are defined as products that contain dietary ingredients, such as vitamins, minerals, herbs, or amino acids, intended to supplement the diet (U.S. Food and Drug Administration, 2005). Globally, DS use is widespread, with prevalence estimates ranging from 40% to over 80%, depending on the population and region. In high-income countries, nearly half of adults and one-third of children report using supplements such as multivitamins, vitamin D, folic acid, and omega-3 fatty acids (Binns et al., 2018; Kim et al., 2013). Reflecting this demand, the global DS market was valued at USD 177.5 billion in 2023 and is projected to reach approximately USD 327.42 billion by 2030 (Grand View Research 2024). In Saudi Arabia, DS use is common, especially among older adults, with herbal remedies also widely practiced (Alharbi et al., 2024; Alhazmi et al., 2023; Alrowais and Alyousefi, 2017). Economically, the DS market in Saudi Arabia was valued at USD 294.7 million in 2021 and is projected to more than double to USD 605.83 million by 2028 (Algaeed et al., 2019; BlueWeave Consulting, 2022). Despite this growth, nutrient deficiencies particularly in vitamin D, iron, and other essential nutrients remain a significant public health concern, especially among older adults. Recent national studies show that 64.3% of Saudi adults are vitamin D deficient and 23.2% experience iron deficiency (Al-Daghri et al., 2022; Hwalla et al., 2017; El-Mouzan et al., 2010).

The use of DSs among older adults, often motivated by the desire to maintain general health, address nutritional deficiencies, or manage chronic conditions. However, this population is particularly vulnerable to adverse interactions due to the high prevalence of polypharmacy and multiple comorbidities (Campos et al., 2024). The concurrent use of DSs alongside prescription medications increases the risk of drug–supplement interactions, which can alter the pharmacokinetic or pharmacodynamic profiles of drugs, potentially resulting in reduced therapeutic efficacy or increased toxicity (Shahverdian and Jafari, 2025; Gomes et al., 2024). In addition, supplement–supplement interactions, arising from the simultaneous use of multiple DSs, may lead to nutrient excesses, deficiencies, or other unexpected adverse effects (Agbabiaka et al., 2018; Zarowitz, 2010). Evidence indicates that older adults who use DSs alongside prescription medications are at risk of potential clinically significant interactions. In the United States, studies have reported risks between 16% and 50%, depending on the population and setting (de Leon et al., 2018; Loya et al., 2009; Jaqua et al., 2024). Similar concerns have been observed in the United Kingdom, where 78% of concurrent users combined DSs with prescription medications and 32.6% were identified as being at risk of adverse interactions (Agbabiaka et al., 2018). The risk is often underestimated, as many patients fail to report their supplement use to healthcare providers (Rask et al., 2004; Fravel et al., 2023). Alarmingly, national data suggest that nearly 15% of older adults faces a high risk of major drug–supplement or drug–drug interactions (Qato et al., 2016). This highlight a critical gap in routine clinical care and emphasize the need for proactive screening and targeted patient education to ensure safer therapeutic outcomes.

Many studies have assessed the pattern, attitudes, and practices related to DS use in Saudi Arabia, highlighting the use across various age groups and regions (Alshehri et al., 2025; Alhashem et al., 2022; Alowais and Selim, 2019; Alfawaz et al., 2019; Aljaloud and Ibrahim, 2013). However, these studies have primarily focused on students or the general population, with limited attention to older adults which a group more likely to use DSs due to comorbidities and age-related nutritional needs. Furthermore, limited studies have evaluated the potential for interactions, one has particularly examined drug–herb interactions among patients with chronic diseases in Saudi Arabia (Albassam et al., 2021). To date, no research has comprehensively evaluated drug–supplement interactions, including both herbal and non-herbal products, among older adults, despite their elevated risk of adverse outcomes. Therefore, this study aimed to assess the prevalence and patterns of DS use, identify associated factors, document reported side effects, and evaluate the prevalence and severity of potential interactions with prescribed medications among older adults in Saudi Arabia.

## 2 Methods

### 2.1 Study design and setting

This was a cross-sectional study conducted among elderly patients (≥60 years) visiting outpatient clinics in Taif, Saudi Arabia.

### 2.2 Questionnaire development

The design of the questionnaire was developed based on previous studies and refined through consultation with an academic expert pharmacist (Agbabiaka et al., 2018; Jermini et al., 2019). The questionnaire consists of 17 questions including four sections as following Section 1: sociodemographic characteristics (6 questions) collected participants’ age, gender, nationality, education level, marital status, and employment status. Section 2: dietary supplement use (5 questions) explored details about dietary supplements, including the types used, frequency of use, dosage, source of recommendations, and place of purchase. Section 3: side effects and reactions (4 questions) gathered information on any adverse events experienced from DS use, including whether participants stopped taking supplements due to these side effects. Section 4: prescription medication use (2 questions) collected data regarding participants’ prescription medications to assess potential drug-supplement interactions.

### 2.3 Pilot questionnaire

The questionnaire was pilot-tested with a sample of 10 participants from the target population to assess clarity, relevance, and the comprehensibility. Feedback from the pilot study was used to refine wording and ensure alignment with the study’s objectives. Data from the pilot study was excluded from the final analysis.

### 2.4 Sample size calculation

The sample size of 377 patients is determined using the Raosoft Sample Size Calculator, targeting a 95% confidence interval, a 5% margin of error, and a 50% response distribution to ensure statistical reliability and precision.

### 2.5 Participant recruitment and data collection

Participants were recruited using convenience sampling methods. Trained researchers conducted structured face-to-face interviews to collect data, using SurveyMonkey to standardize and securely manage responses. Prior to interview, each participant was informed about the study aim and objectives, the voluntary nature of participation, and privacy and confidentiality measures. Data collection took place from November 2024 to March 2025. The inclusion criteria were elderly patients aged ≥60 years who used DSs alongside at least one prescription medications and visited outpatient clinics in Taif.

### 2.6 Assessment of supplement use and interactions

Participants were asked to report all supplements they were taking, including herbal products, vitamins, and minerals, as well as any prescribed medications. Herbal products were excluded from the analysis if they were used exclusively for culinary purposes (e.g., herbs commonly used as spices or flavoring agents). To assess potential interactions between supplements and medications, we used Stockley’s Drug Interactions database which is an authoritative and comprehensive resource for identifying clinically significant drug interactions. Each reported supplements were cross-checked against concurrently used medications to identify any documented or potential interactions. The severity and clinical relevance of the interactions were recorded according to Stockley’s classification (e.g., minor, moderate, or severe), and appropriate notes were taken regarding the nature and potential consequences of these interactions.

### 2.7 Statistical analysis

Descriptive statistics were used to summarize participants’ demographic characteristics, supplement use patterns, and interaction data. Chi-square tests were conducted to assess associations between categorical variables. Univariate logistic regression analyses were used to identify predictors of dietary supplement use and potential drug–supplement interactions. Odds ratios (ORs) with 95% confidence intervals (CIs) were reported. Statistical significance was defined as a p-value <0.05. Data analysis was performed using Stata version 16 (StataCorp LLC, College Station, TX, United States.

### 2.8 Ethical considerations

Ethical approval was obtained from the Research Ethics Committee at Taif University (Approval No.: 46-070, Date: 03-11-2024) and the Scientific Research Ethics Committee at King Faisal Medical Complex in Taif (Approval No.: 2024-E-94, Date: 28-12-2024). Participation was voluntary, and informed consent was secured from all participants. No personally identifiable information was collected, and data was securely stored and accessible only to the research team, ensuring confidentiality and privacy.

## 3 Results

### 3.1 Sociodemographic characteristics

A total of 293 participants were enrolled, of whom 245 (83.6%) reported using DSs. The majority were female (57.7%), and the most represented age group was 60–64 years (34.1%), followed by 65–69 years (27.3%). Most participants were Saudi nationals (97.6%), and nearly half (45.1%) had no formal education. Only 7.5% held a bachelor’s or postgraduate degree. Most participants were married (74.1%), while 21.5% were widowed. The most common chronic conditions were diabetes (60.5%) and hypertension (60.1%), with 8.9% reporting no chronic illnesses.

Significant associations were observed between DS use and several variables. Females were more likely than males to use DSs (88.2% vs. 77.4%, p = 0.014). DS use was highest among those with no formal education (89.4%) and lowest among those with higher education (70.2%) (p = 0.049). Hypertension was also significantly associated with DS use (89.0% vs. 69.6% in those without any condition, p = 0.008), while arthritis was associated with lower use (p = 0.045). Detailed sociodemographic and clinical characteristics and their associations with DS use are presented in Table 1.

**TABLE 1:** Sociodemographic characteristics of participants and their associations (n = 293).

Characteristic		Total n (%)	User of DSs n (%)	Non-user of DSs n (%)	P-value
Gender					
Male	124 (42.32%)		96 (77.42%)	28 (22.58%)	0.016
Female	169 (57.68%)		149 (88.17%)	20 (11.83%)	
Age					
60–64	100 (34.13%)		80 (80.00%)	20 (20.00%)	0.121
65–69	80 (27.30%)		66 (82.50%)	14 (17.50%)	
70–74	63 (21.50%)		59 (93.65%)	4 (6.35%)	
75–79	22 (7.51%)		19 (86.36%)	3 (13.64%)	
80+	28 (9.56%)		21 (75.00%)	7 (25.00%)	
Nationality					
Saudi	286 (97.61%)		239 (83.57%)	47 (16.43%)	0.879
Non-Saudi	7 (2.39%)		6 (85.71%)	1 (14.29%)	
Educational Level					
No formal Education	132 (45.05%)		118 (89.39%)	14 (10.61%)	0.049
Primary and Intermediate Education	72 (12.258%)		54 (74.825%)	18 (25.175%)	
Secondary Education	30 (10.24%)		23 (76.67%)	7 (23.33%)	
Diploma or vocational training	15 (5.12%)		11 (73.33%)	4 (26.67%)	
Bachelor’s Degree or Postgraduate Degree	44 (7.505%)		39 (70.24%)	5 (29.76%)	
Marital Status					
Single	3 (1.02%)		2 (66.67%)	1 (33.33%)	0.557
Married	217 (74.06%)		183 (84.33%)	34 (15.67%)	
Divorced	10 (3.41%)		7 (70.00%)	3 (30.00%)	
Widow	63 (21.50%)		53 (84.13%)	10 (15.87%)	
Medical Condition Status					
I do not have any condition	23 (8.91%)		16 (69.57%)	7 (30.43%)	0.058
Hypertension	155 (60.08%)		138 (89.03%)	17 (10.97%)	0.009
Diabetes	156 (60.47%)		134 (85.90%)	22 (14.10%)	0.261
Cardiovascular disease	66 (25.58%)		58 (87.88%)	8 (12.12%)	0.288
Kidney disease	8 (3.10%)		7 (87.50%)	1 (12.50%)	0.764
Liver disease	3 (1.16%)		3 (100.0%)	0 (0.00%)	0.441
Asthma	11 (4.26%)		8 (72.73%)	3 (27.27%)	0.320
Arthritis	18 (6.98%)		12 (66.67%)	6 (33.33%)	0.045
Osteoporosis	17 (6.59%)		16 (94.12%)	1 (5.88%)	0.228
Other	58 (19.681%)		50 (83.711%)	8 (16.288%)	

### 3.2 Patterns and determinants of dietary supplements use

**FIGURE 1:**
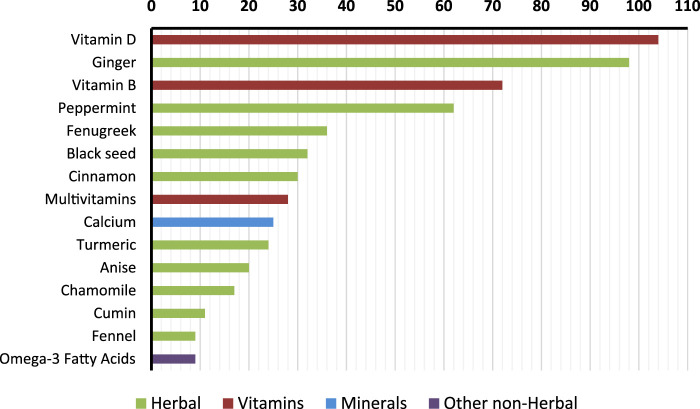
Most frequently reported dietary supplements among older adults, categorized by type.

Among DS users (n = 245), vitamins were the most frequent category, with vitamin D used by 42.4% of participants, vitamin B by 29.4%, and multivitamins by 11.4%. Minerals included calcium (10.2%) and iron (1.2%). Other non-herbal supplements were less common, with omega-3 fatty acids reported by 3.7%. In terms of herbal products, ginger was the most common (40.0%), followed by peppermint (25.3%), fenugreek (14.7%), black seed (13.1%), and cinnamon (12.2%). Figure 1 illustrates the frequency and type of the top reported supplements.

The most common frequency of use was weekly (38.4%), followed by daily (30.5%) and occasional use (25.3%). Common reasons for use included general health (28.3%), vitamin deficiencies (14.5%), and bone/muscle health (13.9%). Doctors (41.2%) and personal decisions (37.6%) were the leading sources of recommendation. Pharmacies (44.6%) and herbalist shops (38.3%) were the most reported sources of purchase. Table 2 summarizes the patterns of DSs use among participants.

**TABLE 2:** Frequency of supplement use, reasons for use, source of recommendation, and purchase locations among participants.

Category and response options	Frequency (n)
Frequency of use	
Daily	195 (30.5%)
Weekly	246 (38.4%)
Monthly	37 (5.8%)
Occasionally	162 (25.3%)
Reason for use	
For general health	181 (28.3%)
For vitamin deficiencies	93 (14.5%)
For bone and muscle health	89 (13.9%)
To improve stomach health	60 (9.4%)
To boost immunity	46 (7.2%)
To support nervous system health	31 (4.8%)
To improve heart and vascular health	26 (4.1%)
I’m not sure	29 (4.5%)
Others (e.g., mood, glucose control, sleep)	85 (13.3%)
Source of recommendation	
Doctor	150 (41.2%)
Personal decision	137 (37.6%)
Family or friends	50 (13.7%)
Pharmacist	11 (3.0%)
Botanist/Herbalist	9 (2.5%)
Media or advertisements	7 (1.9%)
Place of purchase	
Pharmacy	169 (44.6%)
Botanist/herbalist	145 (38.3%)
Supermarket	61 (16.1%)
Health and beauty stores	4 (1.1%)

### 3.3 Adverse effects and discontinuation of dietary supplements

Overall, 15.5% of participants (n = 38) reported adverse effects from DSs, mostly gastrointestinal symptoms (8.4%) such as nausea, upset stomach, and heartburn. Ginger was most frequently implicated (3.2%), followed by multivitamins (2%) and vitamin D, iron, and calcium (1.2% each). A total of 20 participants (12.8%) discontinued one or more supplements due to side effects. The most commonly discontinued products were ginger, liquorice, peppermint, and multivitamins. A summary of the reported side effects and the products discontinued due to these reactions is provided in Table 3.

**TABLE 3:** Reported side effects and discontinuation of dietary supplements among older adult participants.

Dietary supplements	Reported side effects	Discontinued (Yes/No)	Reason for discontinuation
Ginger	Headache, dizziness, insomnia, palpitations, burning, heartburn, low blood glucose, stomach upset	Yes	Stomach upset
Multivitamins	Stomach upset, renal cyst, weight gain, heat sensation	Yes	Stomach upset, renal cyst, weight gain, heat
Vitamin D	Stomach upset, heartburn, dizziness	Yes	Stomach upset
Iron	Constipation, stomach upset, indigestion, feeling unwell	Yes	Constipation, indigestion, lack of expected effect
Calcium	Abdominal pain, kidney pain, nausea, heartburn, difficulty swallowing	No	–
Magnesium	Sleepiness	Yes	Sleepiness
Vitamin B	Nausea, tingling	No	–
Bay laurel	Nausea	No	–
Chamomile	Sleepiness	No	–
Cinnamon – Turmeric	Heartburn	Turmeric: Yes	Heartburn (Turmeric)
Clove – Liquorice	Increased blood pressure	Yes	Increased blood pressure
Common sage	Sleepiness, dizziness, dry mouth	Yes	Sleepiness, dizziness, dry mouth
Cumin	Weight loss	No	–
Fenugreek	Diarrhoea, odour	Yes	diarrhoea, smell
Hibiscus	Dizziness, feeling unwell	Yes	Dizziness, feeling unwell
Peppermint	Stomach upset	Yes	Stomach upset, increased pain, irritation

### 3.4 Prevalence and patterns of drug–supplement and supplement–supplement interactions

Among the 245 DS users, a total of 606 supplements and 593 medications were reported, with averages of 2.61 supplements and 2.56 medications per participant. Interaction analysis was conducted on 232 participants using both medications and DSs. A total of 199 potential interactions were identified, 167 drug–supplement interactions (mean = 0.72 per participant; maximum = 4) and 32 supplement–supplement interactions (mean = 0.14 per participant; maximum = 3). At least one potential drug–supplement interaction was observed in 40.1% (n = 93) of participants, and 10.3% (n = 24) had at least one supplement–supplement interaction. Of the 199 interactions identified, 163 (82%) were moderate, 24 (12%) were severe, and 12 (6%) were mild in clinical significance.

### 3.5 Details and severity of common drug–supplement interactions

After grouping medications by therapeutic class, nicotinic acid emerged as the supplement most frequently involved in potential interactions across multiple drug classes (Table 4). The highest number of interactions occurred with antidiabetic medications, including Metformin (39 interactions), Insulin (26), and Sulfonylureas such as Gliclazide (14). Additional interactions were reported with DPP-4 inhibitors (5) and SGLT2 inhibitors (5), all of which were classified as moderate in severity and required close monitoring of glycemic control. Nicotinic acid also demonstrated severe interactions with statins (16 interactions), such as Atorvastatin and Rosuvastatin, due to the risk of myopathy. Moderate interactions were identified with Aspirin (11 interactions) and Warfarin (3), while severe interactions were reported with Omega-3 fatty acids (3), highlighting potential bleeding risks.

**TABLE 4:** Most frequently identified potential interactions between dietary supplements and medication groups among older adults. Severity levels and recommended actions are based on Stockley’s Drug Interactions database.

Supplement	Medication group	Severity	Recommended action	No. of interactions
Nicotinic acid	Metformin	Moderate	Diabetic control should be closely monitored, recognising that some adjustment of the antidiabetic drugs may be needed	39
Nicotinic acid	Insulin	Moderate	Diabetic control should be closely monitored, recognising that some adjustment of the antidiabetic drugs may be needed	26
Vitamin D	Calcium^a^	Moderate	Monitor calcium levels more frequently if both drugs are considered necessary	19
Nicotinic acid	Statins	Severe	Use the lowest statin dose, or consider dose reduction. Patients should report any unexplained muscle pain or weakness. Caution advised with ≥1 g niacin	16
Nicotinic acid	Gliclazide	Moderate	Diabetic control should be closely monitored, recognising that some adjustment of the antidiabetic drugs may be needed	14
Nicotinic acid	Aspirin	Moderate	The general importance of this interaction is unknown, but it is expected to be limited	11
Calcium	Amlodipine	Moderate	If calcium-channel blocker effects are diminished, monitor serum calcium concentrations	10
Peppermint	Proton Pump Inhibitors	Moderate	Separate administration by several hours; some suggest avoiding concurrent use	7
Ascorbic acid	Aspirin	Mild	Clinical relevance is uncertain. Ascorbic acid requirements may increase in the presence of aspirin	7

^a^ This is a supplement–supplement interaction, not a drug–supplement interaction.

Vitamin D was involved in 29 interactions, mainly with Calcium and Thiazide diuretics. Calcium was involved in 19 interactions, particularly with Amlodipine, Zinc and Iron. Peppermint was associated with 14 interactions, including proton pump inhibitors, Iron, and Calcium. Omega-3 fatty acids were implicated in interactions with Aspirin, Celecoxib, Beta-blockers, and Nicotinic acid. In terms of severity, most interactions were moderate, while 12 interactions were classified as severe, including combinations such as Ginger and Warfarin, Nicotinic acid and Statins. A full list of identified interactions, including medication classes, severity ratings, and recommended actions, is provided in Supplementary Material 1.

### 3.6 Logistic regression analyses of factors associated with dietary supplement use and drug–supplement interactions

Univariate logistic regression analyses were conducted to identify factors associated with DS use and potential drug–supplement interactions among older adults. Female gender (OR = 2.17; 95% CI: 1.16–4.07; p = 0.016), age 70–74 years (OR = 3.69; 95% CI: 1.20–11.36; p = 0.023), and hypertension (OR = 2.35; 95% CI: 1.24–4.47; p = 0.009) were significantly associated with greater odds of DS use. In multivariable analysis, these associations remained significant for female gender (aOR = 2.49; 95% CI: 1.14–5.44; p = 0.022), age 70–74 years (aOR = 3.71; 95% CI: 1.11–12.37; p = 0.033), and hypertension (aOR = 2.30; 95% CI: 1.10–4.80; p = 0.026).

Among DS users, diabetes (OR = 3.67; 95% CI: 2.06–6.54; p < 0.001) and hypertension (OR = 2.34; 95% CI: 1.34–4.09; p = 0.003) were significantly associated with the likelihood of drug–supplement interactions. In multivariable analysis, these associations remained significant for diabetes (aOR = 3.53; 95% CI: 1.88–6.62; p < 0.001) and hypertension (aOR = 2.53; 95% CI: 1.32–4.85; p = 0.005). Additionally, participants aged 65–69 years had lower odds of drug–supplement interactions (aOR = 0.38; 95% CI: 0.17–0.88; p = 0.023). No other sociodemographic or clinical factors were significantly associated with either outcome in the univariate or multivariable models (Table 5).

**TABLE 5:** Univariate and multivariable logistic regression analyses of predictors associated with dietary supplement use and potential drug–supplement interactions among older adults.

	Dietary supplement use				Potential drug–supplement interactions			
Predictor	cOR (95% CI)	p-value	aOR (95% CI)	p-value	cOR (95% CI)	p-value	aOR (95% CI)	p-value
Gender								
Male	1		1		1		1	
Female	2.17 (1.16–4.07)	0.016	2.49 (1.14–5.44)	0.022	0.65 (0.38–1.12)	0.119	0.94 (0.45–1.98)	0.876
Age group								
60–64	1		1		1		1	
65–69	1.18 (0.55–2.51)	0.670	1.29 (0.56–2.98)	0.551	0.67 (0.33–1.35)	0.260	0.38 (0.17–0.88)	0.023
70–74	3.69 (1.20–11.36)	0.023	3.71 (1.11–12.37)	0.033	0.84 (0.41–1.70)	0.619	0.62 (0.27–1.40)	0.248
75–79	1.58 (0.43–5.88)	0.493	1.26 (0.30–5.41)	0.752	1.23 (0.45–3.40)	0.690	0.78 (0.24–2.52)	0.681
80+	0.75 (0.28–2.01)	0.567	0.45 (0.14–1.51)	0.197	1.67 (0.62–4.54)	0.314	1.38 (0.44–4.35)	0.581
Educational Level								
No formal Education	2.57 (0.93–7.05)	0.068	1.85 (0.56–6.08)	0.313	0.86 (0.34–2.16)	0.752	0.68 (0.22–2.07)	0.500
Primary and Intermediate	0.91 (0.34–2.48)	0.859	0.69 (0.23–2.03)	0.498	1.10 (0.41–3.00)	0.846	1.00 (0.33–3.02)	0.996
Secondary	1		1		1		1	
Diploma	0.84 (0.20–3.47)	0.806	1.26 (0.28–5.66)	0.766	0.67 (0.14–3.27)	0.617	0.79 (0.14–4.47)	0.789
Bachelor’s or higher	2.37 (0.67–8.35)	0.178	3.54 (0.95–13.29)	0.061	2.15 (0.72–6.47)	0.172	3.15 (0.92–10.81)	0.069
Medical Condition Status								
Hypertension (yes vs. no)	2.35 (1.24–4.47)	0.009	2.30 (1.10–4.80)	0.026	2.34 (1.34–4.09)	0.003	2.53 (1.32–4.85)	0.005
Diabetes (yes vs. no)	1.43 (0.77–2.65)	0.262	1.39 (0.68–2.82)	0.369	3.67 (2.06–6.54)	<0.001	3.53 (1.88–6.62)	<0.001

## 4 Discussion

This study is the first to investigate the prevalence, patterns, and potential risks associated with DS use among older adults in Saudi Arabia, with particular emphasis on drug–supplement interactions. The findings indicate that DS use is highly prevalent, particularly among women, those with limited formal education and individuals with hypertension. Adjusted models showed that diabetes and hypertension independently increased the likelihood of interactions, underscoring the importance of screening patients with these comorbidities. Vitamin D and Vitamin B were the most commonly used non-herbal products, while ginger and peppermint were the most frequently consumed herbs. Most participants reported concurrent use of supplements with prescribed medications, raising concerns about possible interactions. A substantial proportion also reported side effects primarily gastrointestinal which in some cases led to discontinuation. A wide range of potential drug–supplement and supplement–supplement interactions were identified, many of which were severe or moderate in clinical relevance. Nicotinic acid was frequently implicated in interactions with antidiabetic agents, statins, and antiplatelets, posing risks such as impaired glycaemic control and muscle toxicity. Vitamin D was associated with interactions involving calcium metabolism and cardiac function, while ginger, fenugreek, and omega-3 fatty acids were linked to increased bleeding risk. Additionally, peppermint and calcium were involved in multiple interactions with both medications and other supplements. These findings highlight the need for healthcare providers to routinely assess DS use among older adults and to deliver targeted education to ensure safe, informed, and coordinated care.

The findings of this study indicate that the use of DSs is highly prevalent among older adults in Saudi Arabia, with usage particularly common among women, individuals with limited formal education, and those with hypertension. These trends align with national and international studies reporting similar demographic patterns (Tan et al., 2022; Alhashem et al., 2022). In particular, prior research has shown that women are generally more likely than men to use complementary and alternative medicine, including DSs and herbal remedies, often due to greater health awareness and proactive health behaviors (Bishop and Lewith, 2010; Tindle et al., 2005). Marital status and lower education levels have also been associated with increased reliance on non-prescription products, possibly due to social influence and limited access to formal health education resources (Agbabiaka et al., 2017). Among herbal products, ginger and peppermint were the most frequently used for its anti-inflammatory and gastrointestinal soothing properties, while peppermint is popular for managing symptoms of indigestion and irritable bowel syndrome (Lakhan and Vieira, 2010; Marx et al., 2017). The cultural and traditional familiarity with these herbs may further contribute to their widespread use in Middle Eastern populations.

The findings from this study highlights a significant number of potential interactions between DSs and prescribed medications, several of which may carry clinically important consequences that warrant careful monitoring and management. These findings are consistent with previous research showing that supplement use, especially when combined with prescription medications, can pose serious risks particularly among older adults managing chronic conditions and multiple medications (Agbabiaka et al., 2017; Shahverdian and Jafari, 2025; Campos et al., 2024; Obika, 2022). Broader reviews also emphasize that polypharmacy itself, through both drug–drug and drug–supplement interactions, represents a major contributor to medication-related harm in older adults (Dwivedi et al., 2024). International evidence reinforces this concern, with studies from the United States have reported that between 15% and 50% of older adults using both DSs and prescription drugs were at risk of potential interactions (de Leon et al., 2018; Jaqua et al., 2024; Loya et al., 2009). A survey from the United Kingdom found that 78% of concurrent users combined DSs with prescription medications and nearly one-third were at risk of adverse interactions (Agbabiaka et al., 2018). Age-related physiological changes, including altered drug metabolism and renal function, further increase this population’s susceptibility to adverse interactions (Mangoni and Jackson, 2004). Several of the interactions observed in this study involved commonly used agents such as nicotinic acid, omega-3 fatty acids, vitamin D, and herbal products like ginger. These agents have been previously associated with interactions that may increase bleeding risk, affect glycemic control, disrupt electrolyte balance, or reduce medication efficacy (Posadzki et al., 2013; Hatfield et al., 2022; Hua et al., 2024; Sagers 2010; Thurfah et al. (2022). For example, vitamin D may interact with calcium supplements or thiazide diuretics, potentially leading to hypercalcemia or diminished effectiveness of cardiovascular medications (Robien et al., 2013). If unrecognized, these clinically significant interactions could result in harmful outcomes, particularly in elderly patients with complex medication regimens. Interprofessional collaboration especially between pharmacists and physicians is also crucial in ensuring proper monitoring and educating patients about safe supplement use. In addition to conventional supplements, natural nutraceuticals are increasingly recognized for their complex pharmacological actions. For example, Ursolic acid has been highlighted as carrying potential risks of interacting with conventional therapies, although these effects remain underexplored (Wal et al., 2025). This highlight the need for continued vigilance and further research into supplement safety in older adults.

This study is the first to assess drug–supplement interactions specifically among older adults in Saudi Arabia, providing valuable insights into real-world practices and highlighting potential risks associated with DSs including herbal products. The use of structured face-to-face interviews strengthened data accuracy, particularly given participants’ advanced age and varying literacy levels. Additionally, employing Stockley’s Drug Interactions database improved the clinical relevance of the findings through robust identification of potential interactions. However, this study has several limitations. Convenience sampling may have introduced selection bias, affecting the generalizability of results. The reliance on participants’ self-reporting could have resulted in recall bias, influencing the accuracy of reported data on supplements, medications, and side effects. In addition, because the study relied on self-reported use at a single time-point, intermittent or seasonal use and over-the-counter medications may have been underreported. Moreover, the cross-sectional design precludes causal inference and prevents assessment of the clinical outcomes of the identified interactions, limiting the interpretation of their clinical significance.

Future studies should be conducted at the national level and utilize longitudinal designs to better clarify the causal relationships between DSs use and clinical outcomes. Such longitudinal research would offer deeper insights into the long-term health consequences of drug–supplement interactions, supporting more effective risk identification, prevention, and management strategies. Additionally, future research should evaluate healthcare providers’ awareness, attitudes, and clinical practices regarding dietary supplements, as well as investigate patient education interventions. These studies would facilitate the development of comprehensive, evidence-based guidelines to promote safer and more informed supplement use among older adults nationwide.

## 5 Conclusion

This study highlights the high prevalence of DSs use among older adults in Saudi Arabia, in conjunction with prescribed medications. A substantial number of potential interactions were identified yet may still pose risks such as adverse drug reactions and reduced therapeutic efficacy. Many elderly individuals remain unaware of these risks, emphasizing the critical role of healthcare providers in screening for supplement use and offering informed guidance. Enhancing patient awareness and expanding clinical education on the safety and efficacy of these products are essential. Furthermore, there is an urgent need for robust clinical data to support evidence-based decision-making and improve counselling practices.

## Data Availability

The original contributions presented in the study are included in the article/Supplementary Material, further inquiries can be directed to the corresponding authors.
