# Supplementary material for: Prevalence and patterns of dietary supplement use and potential drug interactions among older adults in Saudi Arabia
Source: Front Pharmacol. 2025 Oct 8;16:1654337. doi: 10.3389/fphar.2025.1654337 (PMC12540427; doi:10.3389/fphar.2025.1654337)
Supplement: Supplementary file 1 [file Supplementaryfile1.docx]

**Supplementary file 1:**

**Table S1: Classification of supplement-medication interactions**

| Supplement | Medication Group | Severity | Action | No. of Interactions |
| --- | --- | --- | --- | --- |
| Nicotinic acid | Metformin | Moderate | Diabetic control should be closely monitored, recognising that some adjustment of the antidiabetic drugs may be needed. | **39** |
| Nicotinic acid | Insulin | Moderate | Diabetic control should be closely monitored, recognising that some adjustment of the antidiabetic drugs may be needed. | **26** |
| Nicotinic acid | Statins | Severe | The lowest statin dose should be used, or a statin dose reduction should be considered. All patients should be warned to report promptly any unexplained muscle aches, tenderness, cramps, stiffness or weakness. Note, that the US manufacturer of nicotinic acid recommends caution with doses of nicotinic acid of 1 g or more on the concurrent use of statins. | **16** |
| Nicotinic acid | Gliclazide | Moderate | Diabetic control should be closely monitored, recognising that some adjustment of the antidiabetic drugs may be needed. | **14** |
| Nicotinic acid | Aspirin | Moderate | The general importance of this interaction is unknown, but it is expected to be limited. | **11** |
| Calcium | Amlodipine | Moderate | There seems no reason to avoid the use of calcium in patients taking calcium-channel blockers, but, if the effects of the calcium-channel blocker are diminished, consider monitoring serum calcium concentrations. | **10** |
| Peppermint | Proton Pump Inhibitors (PPIs) | Moderate | Separate administration by several hours; some suggest avoiding concurrent use. | **7** |
| Ascorbic acid | Aspirin | Mild | The clinical importance of this is uncertain. It has been suggested that the normal physiological requirement of 30 to 60 mg of ascorbic acid daily may need to be increased to 100 to 200 mg daily in the presence of aspirin. | **7** |
| Nicotinic acid | DPP-4 inhibitors | Moderate | Diabetic control should be closely monitored, recognising that some adjustment of the antidiabetic drugs may be needed. | **5** |
| Nicotinic acid | SGLT2 inhibitors | Moderate | Diabetic control should be closely monitored, recognising that some adjustment of the antidiabetic drugs may be needed. | **5** |
| Vitamin D | Thiazide diuretics | Moderate | A clinically relevant interaction seems unlikely in those taking a thiazide with occasional or small doses of vitamin D. Monitor calcium levels in those given a thiazide and high doses of vitamin D regularly. Note that calcium monitoring is a standard recommendation with some vitamin D analogues given alone. | **5** |
| Nicotinic acid | Warfarin | Moderate | Monitor prothrombin times and platelet counts, and adjust treatment accordingly. | **3** |
| Vitamin D | Corticosteroid | Moderate | Monitor calcitriol efficacy. | **3** |
| Omega-3 Fatty Acids | Bisoprolol | Moderate | Bear this potential interaction in mind if blood pressure becomes too low. | **2** |
| Calcium | Alendronate | Moderate | Alendronate should be taken after an overnight fast and patients should wait at least 30 minutes after taking alendronate before taking any other drug or food, and alendronate should be taken with plain water only. | **1** |
| Fenugreek | Warfarin | Moderate | Bear this interaction in mind in the event of an unexpected response to treatment. | **1** |
| Ginger | Warfarin | Severe | Bear this interaction in mind in the event of an unexpected response to treatment. Note that ginger alone does not appear to affect platelet aggregation or coagulation. | **1** |
| Nicotinamide | Carbamazepine | Moderate | Concurrent use should be monitored if large doses of nicotinamide are used. It seems unlikely that small doses (as in multivitamin preparations) will interact to any great extent. | **1** |
| Nicotinic acid | Thiazolidinediones | Moderate | Diabetic control should be closely monitored, recognising that some adjustment of the antidiabetic drugs may be needed. | **1** |
| Omega-3 Fatty Acids | Aspirin | Severe | Warn patients to report any signs or symptoms of bleeding. Consider gastroprotection, such as a proton pump inhibitor. | **1** |
| Omega-3 Fatty Acids | Atenolol | Moderate | Bear this potential interaction in mind if blood pressure becomes too low. | **1** |
| Omega-3 Fatty Acids | Celecoxib | Severe | Warn patients to report any signs or symptoms of bleeding. Consider gastroprotection, such as a proton pump inhibitor. | **1** |
| Vitamin D | Digoxin | Severe | Monitor serum calcium concentrations. Be alert for an increase in digoxin adverse effects (bradycardia, nausea, vomiting), consider monitoring serum digoxin concentrations and adjust the dose as needed. | **1** |
| Iron | Proton Pump Inhibitors (PPIs) | Moderate | Many factors and disease states can affect oral iron absorption. However bear these reports in mind should a patient taking any proton pump inhibitor fail to respond to oral iron therapy. | **1** |
| Iron | Captopril | Mild | No action needed. | **1** |
| Pyridoxine | Nitrofurantoin | Severe | Information appears to be limited to this report, but bear it in mind in the event of unexpected toxicity. | **1** |

**Table S2: Classification of supplement-supplement interactions**

| Supplement | Supplement | Severity | Action | No. of Interactions |
| --- | --- | --- | --- | --- |
| Vitamin D | Calcium | Moderate | Monitor calcium levels more frequently if both drugs are considered necessary. | **19** |
| Calcium | Peppermint | Moderate | Separate administration by several hours. | **4** |
| Nicotinic acid | Omega-3 Fatty Acids | Severe | Monitor for signs and symptoms of bleeding. | **3** |
| Calcium | Iron | Moderate | Calcium carbonate and calcium acetate (in phosphate-binding doses) caused a modest reduction in the absorption of iron from ferrous sulfate. Smaller doses of calcium (e.g. in multivitamin supplements) appear unlikely to have a clinically significant effect. All iron compounds would be expected to be similarly affected. | **2** |
| Calcium | Zinc | Moderate | The clinical importance of this interaction is unknown, but it would seem prudent to separate the administration of zinc from the administration of any calcium salts. Two to three hours separation is often sufficient to achieve maximal absorption with absorption interactions like this. | **2** |
| Zinc | Iron | Moderate | An interaction is not established. However bear the possibility of an interaction in mind should a patient taking iron and zinc fail to respond to either supplement. | **2** |
| Peppermint | Iron | Moderate | The general importance of these findings is uncertain, but be aware that peppermint tea consumption may contribute to iron-deficiency anaemia. However, no restrictions are likely to be needed in healthy patients not at risk of iron deficiency. | **1** |
| Vitamin D | Magnesium | Moderate | The manufacturers say that concurrent use should be avoided. If both drugs are given, monitor magnesium levels. | **1** |
